# Biomaterials and Nanotherapeutics for Enhancing Skin Wound Healing

**DOI:** 10.3389/fbioe.2016.00082

**Published:** 2016-10-31

**Authors:** Subhamoy Das, Aaron B. Baker

**Affiliations:** ^1^Department of Biomedical Engineering, University of Texas at Austin, Austin, TX, USA; ^2^Institute for Biomaterials, Drug Delivery and Regenerative Medicine, University of Texas at Austin, Austin, TX, USA; ^3^Institute for Cellular and Molecular Biology, University of Texas at Austin, Austin, TX, USA; ^4^Institute for Computational Engineering and Sciences, University of Texas at Austin, Austin, TX, USA

**Keywords:** biomaterials, nanoparticles, nanotherapeutics, regenerative medicine, wound care, wound healing, wound dressings, wounds

## Abstract

Wound healing is an intricate process that requires complex coordination between many cell types and an appropriate extracellular microenvironment. Chronic wounds often suffer from high protease activity, persistent infection, excess inflammation, and hypoxia. While there has been intense investigation to find new methods to improve cutaneous wound care, the management of chronic wounds, burns, and skin wound infection remain challenging clinical problems. Ideally, advanced wound dressings can provide enhanced healing and bridge the gaps in the healing processes that prevent chronic wounds from healing. These technologies have great potential for improving outcomes in patients with poorly healing wounds but face significant barriers in addressing the heterogeneity and clinical complexity of chronic or severe wounds. Active wound dressings aim to enhance the natural healing process and work to counter many aspects that plague poorly healing wounds, including excessive inflammation, ischemia, scarring, and wound infection. This review paper discusses recent advances in the development of biomaterials and nanoparticle therapeutics to enhance wound healing. In particular, this review focuses on the novel cutaneous wound treatments that have undergone significant preclinical development or are currently used in clinical practice.

## Introduction

**Figure 1:**
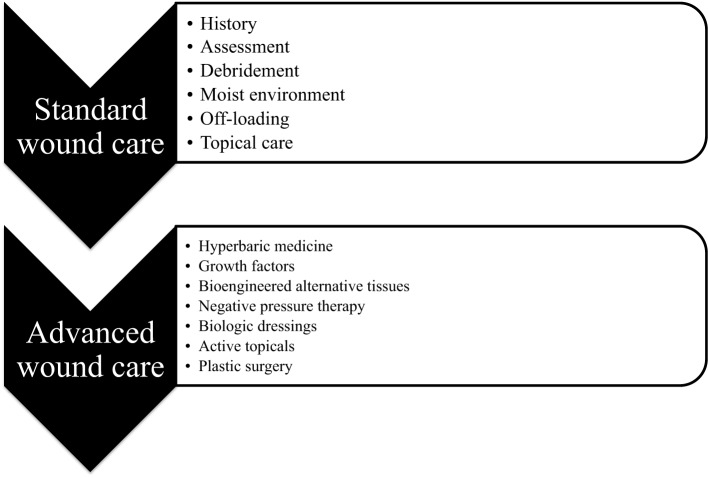
**Clinical wound care**. Schematic diagram showing the differences between standard wound care that is available at any clinic and advanced wound care that is available only in special wound care units across the country.

Cutaneous injuries are a universal aspect of medical care, with approximately 300 million chronic and 100 million traumatic wound patients worldwide. Wounds have an immense financial burden on health-care systems worldwide, accounting for over $25 billion every year in the US alone (Sen et al., 2009). In addition, the incidence of chronic wounds has rapidly increased due to the rising prevalence of type 2 diabetes, peripheral vascular disease, and metabolic syndrome. Although treatments for acute and small area traumatic wounds are effective, problems arise in the long-term care for patients with large area burns, infected wounds, and chronic wounds. Figure 1 provides a summary of the standard wound care procedures present in many wound care clinics and advanced wound care centers, which are present only in specialized wound care units. Clearly, the need for postsurgical and emergency wound care is on the rise, with new treatments being added to the advanced wound care. However, many mechanistic aspects of wound repair remain poorly understood, we direct the reader to other reviews for further information about detailed mechanisms of wound healing (Martin, 1997; Gurtner et al., 2008; Eming et al., 2014). In addition, we refer the reader to reviews of gene and stem cell therapies for wound healing (Branski et al., 2009; Heublein et al., 2015), role of mechanical forces in wound healing (Agha et al., 2011; Wong et al., 2011a, 2012), and immune response to biomaterial implants (Franz et al., 2011) for further information on those topics. In this review, we will focus on biomaterial- and nanoparticle (NP)-based wound therapeutics, in particular, those that have significant preclinical development or are in clinical trials/clinical use. To provide perspective for these therapies, we will first discuss important aspects of healing infection wounds, burn wounds, and chronic wounds, as these types of wounds would benefit most from improved therapies for wound healing.

The prevalence of chronic wounds has increased rapidly in the past decades, creating a major escalation in health-care costs and patient morbidity (Sen et al., 2009). The chronic wounds are heterogeneous in their presentation and etiology. Many times there is high protease activity, large-scale infection and biofilm formation, ischemia or hypoxia in the tissue, recurrent injury due to neuropathy, or cellular failure leading to gangrene associated with chronic wounds. Chronic wounds most often occur as venous leg ulcers, pressure ulcers, or foot ulcers and are found at elevated rates in patients with diabetes and obesity. The prevalence of type 2 diabetes has increased dramatically in the past years and further drives the problem of chronic wound development (American Diabetes, 2013; Futrega et al., 2014). In addition, neuropathy and microvascular angiopathy are common complications of diabetes and contribute to a 12–25% lifetime risk of developing diabetic ulcers (Singh et al., 2005). Specifically, diabetic foot ulcers are responsible for 25–50% of the total cost of diabetes treatment (Armstrong et al., 2013) and are the most common cause for limb amputations in the US (Larsson et al., 1998). Diabetic ulcer is a complex clinical problem requiring a multifaceted treatment plan with standard therapeutic components, including debridement of necrotic tissue, offloading, infection control, surgical revascularization, and limb elevation/compression (Ayello, 2005; Alexiadou and Doupis, 2012; Andrews et al., 2015). Skin grafts, including bioengineered or artificial skin, autografts, allografts, or xenografts from animal tissue, have produced promising results in clinical trials for diabetic foot ulceration (Santema et al., 2016). While many bioactive approaches have been explored to improve wound care in diabetic patients (Calderini et al., 2014), many of these have been found to be ineffective in clinical trials, and these non-healing wounds remain a major clinical challenge (Steed et al., 1992; Richard et al., 1995; Wieman et al., 1998; Steed, 2006; Uchi et al., 2009; Marti-Carvajal et al., 2015).

Infection control is extremely important due to rising incidence of multidrug-resistant microbes all over the world. The widespread use of antibiotics over the past 20 years has led to the development of antibiotic resistance in bacteria and fungi (Bell et al., 2014). The situation is particularly severe in developing countries where there is little control over the sale and usage of antibiotics. Rampant use of antibiotics has selected for the introduction of bacterial genes from the soil “resistome” into human pathogens, as demonstrated by the presence of the same genes in soil bacteria and human bacteria (Blair et al., 2015). *Staphylococcus* is the most common infection found in burns and wounds (Dhanalakshmi et al., 2016). Methicillin-resistant *Staphylococcus aureus* (MRSA) is the most common hospital-borne infection affecting millions of patients daily (Dantes et al., 2013). Interestingly, the microbes affecting patients with chronic infected wounds are dependent on the geographic location as well (Banu et al., 2015; Uckay et al., 2015). In May 2015, the World Health Assembly, a subset of World Health Organization adopted a Global Action Plan against antimicrobial resistance, which shows the urgency of this problem (Assembly, 2015). In December 2015, the US congress has approved a $303 million increase in funding to fight this global epidemic of antimicrobial resistance. Antibiotic-resistant organisms present a major problem in infected wounds, and there is an immense need for engineering new therapies using nanotechnology and advanced biomaterial science to address this problem. The development of efficient antimicrobial drugs against the multidrug-resistant microbes that delay wound healing would provide a substantial benefit to patients with chronic wounds from infection.

Burn injuries also present significant challenges in restoring patient functionality and cosmetic repair. Acute burns result in a sudden influx of inflammatory cytokines and growth factors (Evers et al., 2010). Large area burns often lead to complications, including hypertrophic scarring, facial disfigurement, and loss of muscle and function. Tissue perfusion is of paramount importance to improve wound healing by bringing more nutrients and creating hyperoxyic conditions suitable for healing (Ramasastry, 2005). Current therapeutic regimes for burns are painstakingly long and often still result in scarring and contraction that must be addressed through further therapies and long-term care. Effective wound dressings that induce functional reconstruction following burn injury would have a profound impact on patients with large area burns.

The ideal advanced wound dressing would maintain the wound microenvironment and address the limitations of wound healing specific to the type of wound and patient’s accompanying disease or injury state. It is generally advantageous for wound dressings to be breathable, allowing optimal gaseous exchange and thus protect the periwound skin from maceration and assist in autolytic debridement in removing debris and necrotic tissues. The dressing must also maintain the balanced moist wound environment by donating moisture to the dry wounds and absorbing moisture and exudates from wet wounds. It must act as a barrier to protect against infections and provide thermal insulation to the wound. Finally, advanced wound dressings should aim to enhance aspects of the natural wound healing processes. These could include the promotion of angiogenesis in wounds with poor perfusion, modulation of the immune cells within the wound, enhancement of the invasion and migration of fibroblasts and keratinocytes in the healing wound, and prevention or treatment of infection.

In this review, we focus specifically on the current biomaterial or NP-based therapies that have significant preclinical development or have entered into clinical usage or trials. We discuss the Section “Fundamental Aspects of Wound Healing” briefly followed by sections devoted to “Biomaterial-Based Therapies” and “Nanoparticle-Based Wound Therapies.” We chose to focus on both biomaterials- and NPs-based therapies because with the advent of new and advanced technologies, the fields have become interdependent. In our opinion, we need to be cognizant of the advances in both the fields to be able to develop new therapies to tackle the myriad challenges in the field of wound healing.

## Fundamental Aspects of Wound Healing

**Figure 2:**
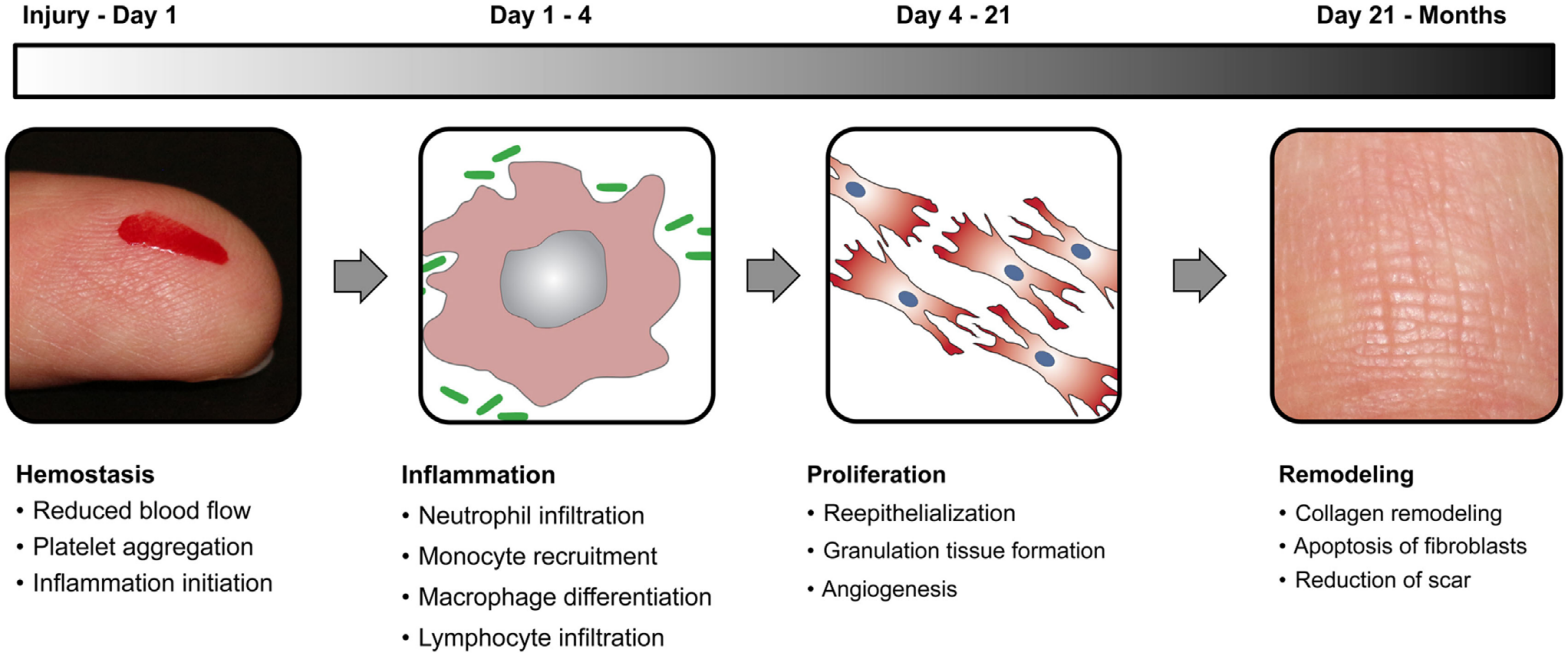
**Wound healing phases**. Schematic diagram elucidating the four distinct stages of normal wound healing, including hemostasis, inflammation, proliferation, and remodeling, along with the time scale of each phase.

There are four relatively distinct phases in wound healing process that include hemostasis, inflammation, proliferation, and remodeling (Figure 2). To understand the process better, let us draw analogy with a kingdom at war. The first step during war is to close the gates of the castle that is equivalent to the hemostasis phase of wound healing, which entails clotting of blood mediated by platelets. The next step is to mount a massive attack against the enemy, which is similar to the inflammation phase of wound healing, mediated by macrophages and neutrophils. Then a huge number of repair people like plumbers, roofers, framers, and laborers are recruited to fix the damage done to the castle, which is similar to the proliferation phase of wound healing, mediated by macrophages, lymphocytes, fibroblasts, and keratinocytes. Finally, the renovation of the castle is handled by interior designers for the final redecoration similar to the remodeling phase of wound healing mediated primarily by fibroblasts. Wound healing phases are in delicate balance with each other, especially the inflammation and proliferation phases. If there is too much inflammation during healing, it leads to chronic non-healing wounds that are common in many peripheral vascular diseases and type 2 diabetes patients. On the contrary, too much proliferation during healing leads to scar formation that is not esthetically pleasing and reduces the quality of life. In addition, if there is damage to the underlying muscle tissue, the satellite cells are activated to form myoblasts to initiate the muscle healing process, which generally takes even longer than skin wound healing.

### Hemostasis Phase

Following the initial wounding, there is the potential for bleeding and requiring hemostasis (Figure 2). On the battlefield, blood loss or hemorrhage from acute injury is the leading cause of deaths for soldiers (Clifford, 2004). Even though tourniquets are still used in the field to stop blood flow temporarily in large wounds, it may lead to ischemia and reperfusion injury in the tissue (Percival and Rasmussen, 2012). Thus, products to enhance hemostasis are of paramount importance in preventing exsanguination and hemorrhagic shock in people with extensive wounds. The most commonly used methods to facilitate hemostasis are direct pressure on the wound and application of hemostatic materials. Hemostatics can be of three different types including clotting facilitators (e.g., kaolin) and mucoadhesive agents (e.g., chitosan) (Kozen et al., 2008).

### Inflammation Phase

The natural response of the immune system to any bodily injury is to monitor the condition and illicit inflammatory reaction to tackle the foreign particles (Figure 2). The classic signs of inflammation have been described since first century AD in Rome and are referred to as dolor (pain), calor (heat), rubor (redness), and tumor (swelling) (Karimbux, 2014). The inflammatory response is mediated by the neutrophils and monocytes (that differentiate into macrophages) (Broughton et al., 2006). The neutrophils are involved in infection control, and macrophages remove cellular debris and provide soluble signals that activate fibroblasts and myofibroblasts in the proliferation phase of wound healing by releasing various kinds of cytokines, proteases, and growth factors in the wound. For patients with chronic conditions, the wounds often acquire a highly inflammatory state, necessitating the use of non-steroidal anti-inflammatory drugs (salicylates, arylalkanoic acids, 2-arylpropionic acids, *N*-arylanthranilic acids, pyrazolidines, oxicams, and COX-2 inhibitors) (Su et al., 2010) and antibiotics (polymyxins, macrolides, tetracyclines, aminoglycosides, lincosamides, streptogramins, and pleuromutilins) (Martin, 1997).

### Proliferation Phase

This is the rebuilding phase of the wound healing process (Figure 2). During the inflammatory phase, the macrophages and neutrophils release various cytokines and chemokines that attract cells into the wound microenvironment, including other lymphocytes, endothelial cells, fibroblasts, myofibroblasts, and keratinocytes (DiPietro, 1995). The keratinocytes migrate from the wound edge, cover the wound bed, and restore barrier function in the skin. The fibroblasts proliferate and secrete various extracellular matrix (ECM) proteins including fibrin, fibronectin, collagen and other ECM proteins that provide a provisional matrix for tissue remodeling and angiogenesis. This forms the granulation tissue of the wound, which is imperative for the proper wound healing (Mayet et al., 2014). Lymphocytes and other immune cells provide additional responses to infectious agents, continuing the processes initiated by the early influx of neutrophils. The endothelial cells under the stimulation of soluble factors released by platelets, macrophages, and other cells initiate neovascularization in the wound bed to improve nutrient and oxygen exchange. The new vessels also aid in the transport of other cells into the affected area facilitating the wound healing process. This step can last anywhere from 4 days to 3 weeks.

### Remodeling Phase

The final phase of the wound healing process is the remodeling of the wound and surrounding tissue by the fibroblasts, which start in about 3 weeks after injury and can continue until as long as 2 years (Figure 2). The fibroblasts and myofibroblasts put down a network of collagen fibers and other ECM proteins in an orderly manner while using proteases to degrade existing disordered tissue. The granulation tissue formed during proliferation phase is made up of immature type III collagen and is relatively weak. During remodeling, the fibroblasts gradually replace the type III collagen with mature type I collagen (Hantash et al., 2008). The final goal is to restore the tissue to pre-injury conditions during which the wound becomes gradually less vascularized.

### Animal Models for Wound Healing

To study wound healing effectively by mimicking the human wound healing process, a number of wound healing animal models (Kim et al., 2015) have been developed in mouse (Wong et al., 2011b), rat (Dorsett-Martin and Wysocki, 2008), rabbit (Chien, 2007; Aksoy et al., 2009; Pelizzo et al., 2015), and pig (Sullivan et al., 2001). Small mammals such as rats, mice, and rabbits are relatively inexpensive, require fewer resources, have multiple mutant models for delayed wound healing, and thus are easily obtainable. Furthermore, the wound healing process in small mammals is completed in 1–2 weeks instead of weeks or months in human clinical studies. However, a major limitation of these models is the differences in the mechanisms of wound healing in comparison to human wound healing and, in particular, the complexities of a chronic non-healing wound (Ansell et al., 2012). The human skin anatomy is significantly different from that of rats, mice, or rabbits (Ansell et al., 2012). Therefore, the wound healing process is vastly different and hence difficult to compare. In addition, rodents primarily use wound contraction using the underlying thin muscle layer, panniculus carnosus to heal wound, while humans heal wounds by granulation tissue formation (Dunn et al., 2013). Among large animal models, porcine skin has a close structural resemblance to human skin in terms of epidermal thickness and dermal-to-epidermal thickness ratio (Sullivan et al., 2001). They also share similar patterns of hair follicles and vasculature in the skin. Moreover, the dermal collagen and dermal elastic content in porcine skin is more similar to humans than other commonly used mammals (Sullivan et al., 2001). However, a major limitation of wound healing models in pigs is the significant costs of housing and care of the animals, variable wound contraction in pigs depending on the location, and the high rate of growth of pigs that can skew the wound healing process.

## Biomaterial-Based Wound Therapies

Biomaterials have been used to enhance wound healing since the earliest medicine-related writings. Ancient writings from the Egyptians include references to the usage of honey, grease, and vegetable fiber for enhancing wound healing (Shah, 2011). Biomaterials have become an integral part of the medical industry since twentieth century. The global biomaterials industry is estimated to be $150 billion and has been growing steadily (Ratner, 2007). After World War II, restricted classes of new polymers were released to the public and were adopted into medical devices. These materials included polyesters, silicones, fluoropolymers, polyurethanes, nylons, and methacrylates (Ratner, 2007). These polymers were then used for biomedical applications, such as vascular grafts, intraocular lenses, hip prostheses, hydrocephalus shunts, kidney dialysis systems, and other medical devices. Currently, the biomaterials used in the medical industry can be broadly classified into three categories: metals (stents, dental implants, etc.), ceramics (orthopedic and dental implants), and polymers (sutures, vascular grafts, joint tissue, soft tissue in general) (Ratner, 1993). Biomaterials have been tremendously important in the wound care industry specifically for wound dressings, cell encapsulation therapies, and NP encapsulation therapies. We will discuss the role of biomaterials in the wound healing applications in this section.

### Biomaterials Currently in Development

A summary of the biomaterials currently in development is shown in Table 1.

**Table 1:** **Biomaterial-based dressings in development**.

Type	Constituent	Therapeutic benefit	Reference
Standalone	Siloxysilane	Non-stinging, spray-on liquid bandages to protect skin from moisture thus preventing maceration	Salamone et al. (2016)
	Dextran	Complete skin and nerve regeneration in porcine burn wound model	Shen et al. (2015), Sun et al. (2011)
	Urethane	Porous tissue scaffold selectively degraded by ROS in the wound site in rat model	Martin et al. (2014)
	Collagen	UV cross-linked collagen–glycosaminoglycan matrices have reduced toxicity compared to glutaraldehyde-based dermal substitutes	Lew et al. (2007)
	Synthetic	Synthetic cell adhesive polypeptide hydrogel with antibacterial activity against *Escherichia coli* JM109 and *Staphylococcus aureus* ATCC25923	Song et al. (2012)
With bioactive components	Fibrin	Engineered ECM super-affinity growth factors induced repair in chronic wounds and bone defects using a diabetic model	Martino et al. (2014)
	Hyaluronic acid	Hydrogel encapsulating AFS cells that could store and release growth factors and cytokines secreted from those cells after the cells were long gone	Skardal et al. (2016)
	Hyaluronic acid	Thin films of polysaccharide-decorated nanoparticles loaded with vitamin E result in controlled release of the vitamin and a reduction in water loss	Pereira et al. (2016)
Cell encapsulating	Poly β amino ester	Genetically edited MSCs, encapsulated in scaffolds, implanted in ischemic mouse model showed enhanced angiogenesis and limb salvage while reducing muscle degeneration and tissue fibrosis	Yang et al. (2010)
	Fibrin and PEG	ASCs embedded in FPEG gels showed enhanced wound healing and angiogenesis in a rat excisional wound model	Zamora et al. (2013)
	PEG + RGD	Injectable, microporous, cell adhesive scaffolds that lead to rapid cutaneous-tissue regeneration in mouse model	Griffin et al. (2015)
Nucleic acid delivering	Collagen	PDGF DNA gene delivery using collagen hydrogels accelerated wound healing in ischemic dermal ulcers in rabbit model	Tyrone et al. (2000)
	Hyaluronic acid	Hyaluronic acid hydrogels that are MMP degradable for localized delivery in the wounds are embedded with VEGF plasmids that enhance wound healing in diabetic mouse model	Tokatlian et al. (2015)
	Polyurethane	siRNA-loaded nanoparticles, embedded in a pH-responsive and biodegradable scaffold that protects siRNA from degradation and leads to local silencing of genes in mouse excisional wound model	Nelson et al. (2013)
	Chitosan, dextran sulfate, and poly 2	Ultrathin polymer coating delivering siRNA targeting MMP-9 gene for 2 weeks enhances wound healing in diabetic mouse model	Castleberry et al. (2015)
Animal product-based	Small intestine submucosa	Prospective, randomized, controlled multicenter clinical trial with SIS demonstrated 55% of wounds heal compared to 34% in standard care	Mostow et al. (2005)
	Amniotic membrane	Bovine lyophilized amniotic membrane extract tested on rabbit ear wound model demonstrating increased epidermal and dermal regeneration compared to control	Kang et al. (2013)
	Fibroin	Silk fibroin and gelatin-based layered wound dressing in a randomized clinical trial of split thickness skin graft model showed significantly less pain and more rapid skin functional barrier recovery	Hasatsri et al. (2015)
	Marine collagen	Composite film of collagen showed significant wound regeneration in a full-thickness wound in the rat dorsal region resulted in enhanced the formation of blood capillaries	Shen et al. (2015)
Drug or antibiotic loaded	Chitosan and PEG	Chitosan microspheres loaded with silver sulfadiazine impregnated in PEGylated fibrin gels exhibit microbicidal activity against *Staphylococcus aureus* and *Pseudomonas aeruginosa*	Seetharaman et al. (2011)
	Carrageenan, polyox, HPMC	Optimized polyox and carrageenan film dressings loaded with streptomycin and diclofenac that targets bacterial infection and inflammatory phase of wound healing	Pawar et al. (2013), Boateng et al. (2013)
	Polyurethane and dextran	PU–dextran–ciprofloxacin loaded nano fibers showing good bactericidal activity against both of Gram +ve and Gram −ve bacteria	Unnithan et al. (2012)
	PEG and chitosan	Ciprofloxacin loaded PEG–chitosan scaffold for quicker and regulated wound healing in a mouse model	Sinha et al. (2012)
	PEG	Prolyl hydroxylase inhibitor loaded in an injectable hydrogel, tested in ear hole punch injury in MRL mice showing enhanced wound healing *via* drug-induced stabilization of hypoxia-inducible factor-1α (HIF-1α) protein	Zhang et al. (2015b)

#### Standalone Biomaterials

**Figure 3:**
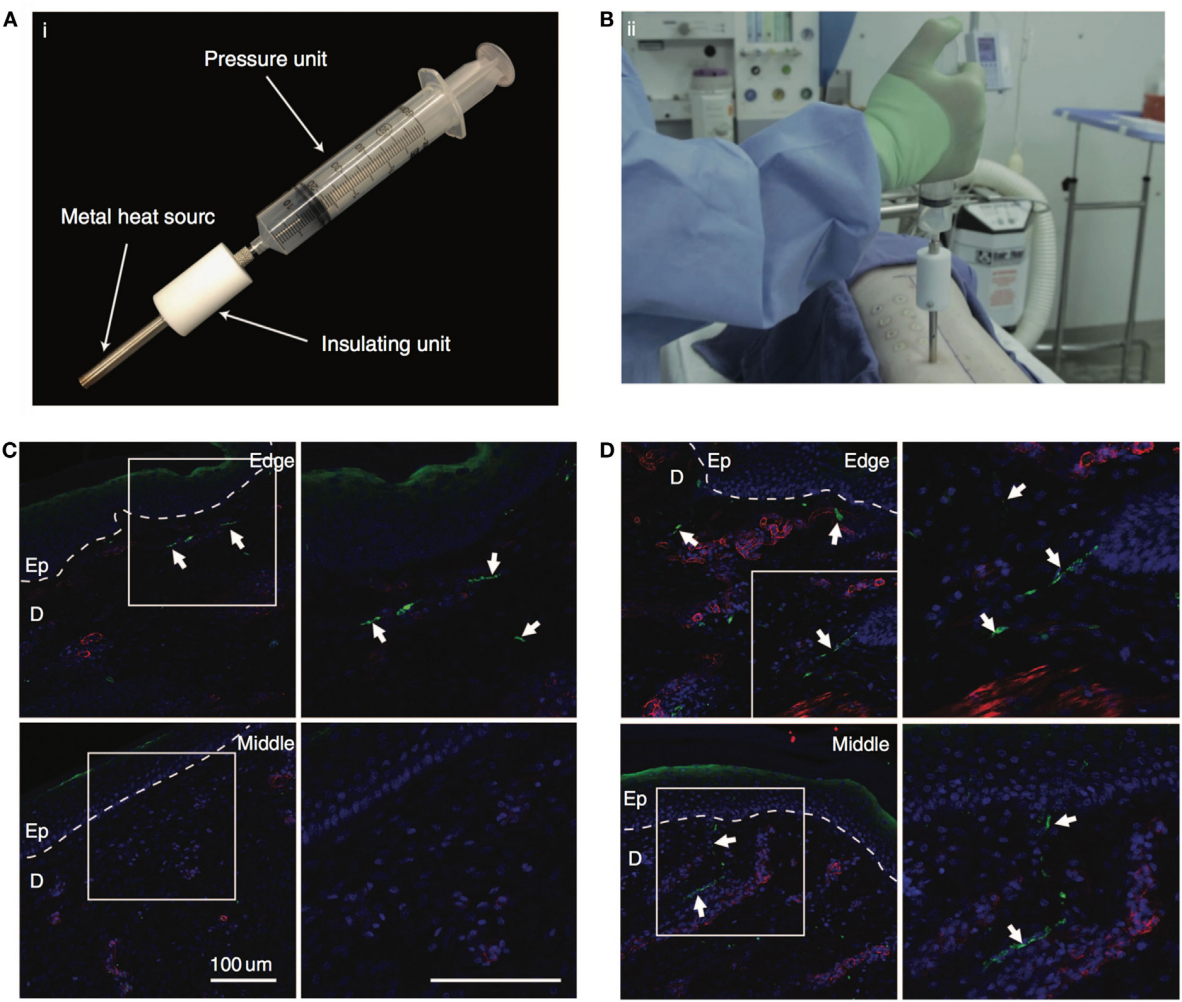
**Nerve regeneration in porcine wound model**. **(A)** The custom-made device with pressure unit to create the burn wounds on the dorsal region of porcine skin. **(B)** The surgeon creating the burn wounds on the thoracic paravertebral region using the custom device. **(C,D)** Immunostaining of the wound tissue at day 40 treated with non-adhesive Curity dressing (Covidien) and dextran hydrogel, respectively. Upper panel shows the tissue edge, and lower panel shows the middle of the tissue. White arrows show the neuronal fibers. Staining for neurons (PGP9.5) is shown in green, blood vessels (α-smooth muscle actin) in red, and nuclei (DAPI) in blue. Bar = 100 μm. Reproduced with permission from Shen et al. (2015).

There has been significant rise in engineering biomaterials for the wound care industry (Salamone et al., 2016). An advanced liquid adhesive bandage has been developed that provides a liquid, amphiphilic, siloxysilane polymer-containing coating material, with or without an antimicrobial agent, that can act as a bandage or coating on skin, on a device or on a dressing to prevent damage to wounds, skin, or mucosal tissue resulting from applied pressure, friction, and shear forces (Salamone et al., 2016). Dextran-based hydrogels have been used in third-degree porcine burn wound model and showed enhanced wound closure, reepithelialization, and nerve reinnervation compared to the control group (Sun et al., 2011; Shen et al., 2015). The most promising feature of dextran-based hydrogels for burn wounds is the efficient nerve regeneration compared to the non-adhesive Curity dressing treatment (Figure 3). Biomaterials that are selectively degraded by reactive oxygen species (ROS) have been used to create wound healing scaffolds with matched rates of tissue in growth- and cell-mediated scaffold biodegradation (Martin et al., 2014). These poly-thioketal urethane-based scaffolds were stable for 25 weeks in aqueous conditions but were degraded by tissue ROS in 7 weeks resulting in enhanced wound closure compared to polyester urethane dressings (Martin et al., 2014). Biomaterials based on bioglasses have recently also shown potential in enhancing wound healing and angiogenesis in animal model of wound healing (Mao et al., 2015; Xu et al., 2015; Zhao et al., 2015; Yu et al., 2016; Zhou et al., 2016). For instance, copper-doped borate bioactive glass microfibers were shown to increase both the rate of collagen deposition and angiogenesis in full-thickness wounds in rats (Zhao et al., 2015). In addition, artificial dermal constructs are also important in healing wound defects. Previous methods of preparation resulted in residual aldehydes left over from the manufacturing process. UV cross-linking has been used to create efficient artificial dermal constructs of collagen and glycosaminoglycan mixed in a hydrogel for wound healing applications (Lew et al., 2007). They tested it *in vitro* with human keratinocytes and found higher cell proliferation and biocompatibility compared to chemically cross-linked constructs. Synthetic hydrogels with variable lengths of polypeptides linked to polyethylene glycol (PEG) have been shown to have both antibacterial and cell adhesive properties (Song et al., 2012). Standalone hydrogels for wound healing is a promising approach and provide a starting point for delivering bioactive agents through direct conjugation or encapsulation as discussed below.

#### Biomaterials Encapsulating Bioactive Components

In the past decades, growth factors were tested as topical treatments to enhance wound healing. However, these growth factor therapies have met with only limited success in clinical trials, and the majority of studies are small- to mid-sized clinical trials that often track only complete wound closure but not percent closure or time to closure, often leaving the clinical recommendation for these therapies unclear. The only approved clinical growth factor treatment for chronic wounds is recombinant platelet-derived growth factor-BB (PDGF-BB) (Becaplermin), and, while approved by the FDA, it has shown mixed results in clinical trials on chronic ulcers and has only seen limited clinical adoption (Fang and Galiano, 2008; Papanas and Maltezos, 2008; Buchberger et al., 2011). Other growth factors, including FGF-2 and epidermal growth factor (EGF), have either shown no improvement or shown only moderate benefits in small clinical trials (Richard et al., 1995; Acosta et al., 2006; Fernandez-Montequin et al., 2009). A structural domain in placenta growth factor-2 (PlGF-2) has been shown to bind strongly to ECM proteins, including fibronectin, vitronectin, tenascin, osteopontin, fibrinogen, and collagen I. By fusing this domain to other growth factors like vascular endothelial growth factor-A (VEGF-A), PDGF-BB, and bone morphogenetic protein-2 (BMP-2), super-affinity growth factors were created (Martino et al., 2014). These engineered growth factors were shown to significantly enhance diabetic wound healing in mice compared to native growth factors. A recent study delivered amniotic fluid-derived stem (AFS) cells, which are known to secrete cytokines and growth factors, in full-thickness skin wounds in a nu/nu murine model (Skardal et al., 2016). The AFS cells have only a limited lifetime *in vivo*, but a photo-crosslinkable heparin-conjugated hyaluronic acid hydrogel preserved the growth factors and cytokines produced by the cells, allowing these paracrine factors to be released into the wound. The treatment improved wound closure with enhanced reepithelialization and increased vascularization and production of ECM (Skardal et al., 2016). Hyaluronic acid-based wound dressings that release α-tocopherols (vitamin E) have been shown to improve wound healing (Pereira et al., 2016). Since the tissue microenvironment has a plethora of chemical cues, delivering of one bioactive component may not be enough to address all the issues of impaired wound healing. In addition, temporal control in release of factors, correlating with the different phases of wound healing, would likely be an important aspect for promoting healing in complex wounds.

#### Biomaterials Encapsulating Cell Therapies

Stem cells or progenitor cells have great regenerative potential especially during injury to the body. In order to target chronic wounds, mesenchymal stem cells (MSCs) were engineered to overexpress the vascular endothelial growth factor (VEGF) gene and encapsulated in a poly(β-amino ester) hydrogel (Yang et al., 2010). Fibrin and PEG gels encapsulating adipose-derived stem cells (ASCs) from discarded burn skin samples were tested in a rat excisional wound model (Zamora et al., 2013). The ASCs significantly improved wound healing outcomes by day 16 compared to the controls. Stem cell therapies delivered in biomaterials have also shown promise for burn wound healing (Ozturk and Karagoz, 2015), diabetic ulcers (Heublein et al., 2015), and cutaneous wound healing (Branski et al., 2009). Microporous annealed particle (MAP) gels were created by mixing pre-gel mixture including a PEG-VS containing an RGD peptide region and matrix metalloproteinase (MMP) substrate with MMP crosslinker solution in a microfluidic channel to make gels that were annealed using FXIIIa into a final microporous scaffold (Griffin et al., 2015). The gels were loaded with dermal fibroblasts and MSCs during *in vitro* studies and found to have robust tubular network formation within 48 h. MAP gels combine the important wound dressing properties of injectability and microporosity that provide mechanical support for rapid cell migration, molecular cues to direct cell adhesion, and resorption after tissue regeneration (Griffin et al., 2015). MAP gels have shown to be effective in wound healing in both *in vitro* and mouse model of excisional wound healing. Encapsulation of newly developed induced pluripotent stem cells (Li and Li, 2014), reprogrammed adult cells, and other stem cells in hydrogels will be interesting while modulating the hydrogel properties to mimic the tissue microenvironment.

#### Nuclei Acid Delivering Biomaterials

A comprehensive genetic study mapping the gene expression profiles during the cutaneous wound healing has motivated the development of gene delivery strategies in facilitating wound healing (Deonarine et al., 2007). Biomaterial-based nucleic acid delivery systems reduce degradation, enhance uptake, and control the treatment dose. Such gene delivery systems can be formulated from biocompatible, biodegradable, and FDA-approved polymers such as poly-l-lactic acid and poly-d,l-lactide-co-glycolide (Kim et al., 2005). Collagen hydrogels encapsulating DNA encoding the PDGF-BB gene is effective in accelerating wound healing in ischemic dermal ulcers in New Zealand white rabbits (Tyrone et al., 2000). Hyaluronic acid-based porous hydrogels containing an MMP-degradable linker that encapsulates the VEGF plasmid DNA results in pro-angiogenic effects and improved diabetic wound healing in mice compared to non-porous hydrogels (Tokatlian et al., 2015). Since PDGF-BB is the only FDA-approved growth factor for diabetic foot ulcers, there have been numerous attempts in enhancing the gene delivery methods for PDGF (Petrie et al., 2005).

The skin microenvironment is teeming with proteases including MMPs, which are significantly upregulated during the wound healing process (Madlener et al., 1998; Steffensen et al., 2001; Rohani and Parks, 2015). These proteases degrade therapeutic proteins like growth factors, cytokines, and chemokines; and ECM proteins including collagen, fibronectin, and vitronectin. Therefore, silencing these proteases has become an important goal in wound healing research. Small interfering RNA (siRNA) can provide gene-specific silencing and present a safe and effective route for knockdown of inflammatory or other target proteins in chronic skin wounds. To modulate the release kinetics and injectability, pH-responsive smart polymer nanoparticles (SPN) are loaded with an injectable polyurethane scaffold (Nelson et al., 2013). The SPNs feature electrostatic loading, nuclease protection of siRNA, and pH-dependent membrane disruptive activity. Polyurethane formulations can be directly injected into a wound or defect where they cure into mechanically robust, biodegradable scaffolds that conform precisely to the shape and size of the wound. In this study, they delivered siRNA against GAPDH along with PDGF-BB encapsulated in the scaffold and showed enhanced excisional wound healing mice (Nelson et al., 2013). Another family of self-assembled wound dressings silence MMP-9 and improve wound healing in diabetic mice (Castleberry et al., 2015). Cationic star-shaped polymers have been used as siRNA carrier for reducing MMP-9 expression in skin fibroblast cells and promoting wound healing in diabetic rats (Li et al., 2014). There are many appealing aspects of delivering RNA-based therapeutics including those using miRNA, lncRNA, piRNA, or shRNA; however, delivering these in an effective manner remains a major objective in the field.

#### Animal Product-Based Biomaterials

Biomaterials derived from natural products can provide materials with greater complexity and composition. In order to mimic the ECM conditions of the wound and to provide a scaffold for the fibroblasts for collagen deposition, ECM-based therapies have gained popularity. The porcine small intestine submucosa that has been lyophilized and sterilized has been recently shown to provide similar benefits to those provided by fish skin-based products for enhancing healing dermal wounds (Mostow et al., 2005). Similarly, lyophilized bovine amniotic membrane has been effective in wound healing applications (Kang et al., 2013). The silk protein, fibroin, is an effective scaffolding material providing a fine mesh for the cells to grow and enhance wound healing (Hasatsri et al., 2015). A phase I clinical trial using fibroin to enhance wound healing is currently underway. Finally, there have been numerous marine polysaccharide hydrogels like marine collagen from *Stomolophus nomurai meleagris, Oncorhynchus keta, Lates calcarifer, Stichopus japonicas*, and *Salmo salar*, alginate from *Macrocystis pyrifera*, chitosan from crabs and shrimps, which are bioactive and increase wound healing rates in mice (Chandika et al., 2015). There is rising need for quality control to prevent transmission of diseases and pathogens from animal products. Lyophilization, pasteurization, and sterilization are important techniques to reduce cross contamination. The major concerns with natural products include batch-to-batch variability, long-term immunogenicity, and safety (Pashuck and Stevens, 2012; Prestwich et al., 2012). Animal product-based biomaterials have been explored only in a limited manner until for chronic wounds, leaving tremendous potential for the development of new therapies in future studies.

#### Antibacterial and Drug-Loaded Biomaterials

A better understanding of the molecular mechanisms underlying the antibiotic resistance will help engineer efficient drugs to target these resistant organisms (Blair et al., 2015). Potential modes of resistance include reduced permeability to antibiotics, increased efflux of antibiotics, alterations in antibiotic targets through mutations, modification or protection of targets, and direct chemical modifications of the antibiotics. Bacterial resistance to antibiotics is inversely correlated with the rate of metabolism, with lower metabolism leading to higher resistance and *vice versa* (Bryan and Van Den Elzen, 1977; Kohanski et al., 2007; Allison et al., 2011; Martinez and Rojo, 2011). Recently, it has been shown that both Gram-negative bacteria and Gram-positive bacteria were killed by kanamycin when the microbes were pretreated with alanine or glucose that promotes the TCA cycle by substrate activation (Peng et al., 2015). Finally, increasing the microbial ROS production makes the *Escherichia coli* susceptible to antibiotics (Brynildsen et al., 2013).

Biomaterial-based wound dressings are ideal for loading drugs or antibiotics due to their tunable properties and release kinetics. Chitosan microspheres loaded with silver sulfadiazine encapsulated in PEG fibrin gels showed robust antimicrobial activity against *S. aureus* and *Pseudomonas aeruginosa* (Seetharaman et al., 2011). Streptomycin has been loaded into polyethylene oxide (PEO) polymer composite films (Pawar et al., 2013), PEO with carrageenan composite films (Boateng et al., 2013), and PEO with alginate composite films (Pawar et al., 2014) for improving wound healing. Similarly, ciprofloxacin has been loaded into electrospun polyurethane and dextran dressings (Unnithan et al., 2012) and PEG–chitosan scaffold (Sinha et al., 2012). Many other antibiotic agents have been loaded into various natural, synthetic, or composite wound dressings. There is a revived interest, and recent academic and industrial research spending has increased with the goal of developing efficient and targeted antimicrobial drugs that have enhanced uptake, reduced degradation, and metabolic mimicry to increase uptake. Apart from antimicrobials, many small molecule drugs and intermediates have been delivered using biomaterial-based systems (Hubbell, 1996). The drug 1,4-dihydrophenonthrolin-4-one-3-carboxylic acid (1,4-DPCA), which is a prolyl hydroxylase (PHD) inhibitor was loaded in a locally injectable hydrogel to achieve controlled delivery of the drug over 4–10 days (Zhang et al., 2015b). This stabilized the constitutive expression of hypoxia-inducible factor-1α (HIF-1α) protein, an important factor in wound healing and angiogenesis.

### Biomaterials in Clinical Usage

A summary of the biomaterials currently in clinical usage is shown in Table 2.

**Table 2:** **Biomaterial-based dressings in clinical usage**.

Type	Constituent	Examples	Indications
Films	Polyurethane	Tegaderm, Blisterfilm, ClearSite, Comfeel film, Suresite, Procyte, OpSite, Dermaview	Minor burns, pressure areas, donor sites, postoperative wounds, and various minor injuries including abrasions and lacerations
Hydrogels	Glycerin	BIolex, elastogel, Curasol gel, Elasto-Gel, flexigel, IntraSite gel, Restore Gel, Hypergel, tenderwet, SoloSite, Vigilon	Necrotic or dry ulcers
Wafers	Hydrocolloids	DuoDERM, Restore plus, RepliCare, Exuderm, Tegasorb, DuoFilm, Cutinova Hydro, nuderm	Mildly exuding ulcers
Foams	Polyurethane	Lyofoam, PolyMem, COPA, Optifoam, Gentleheal, Allevyn	Heavily exuding ulcers, granulating ulcers, painful ulcers
Hydrogels	Alginate	Calcicare, nuderm, SeaSorb, Sorbsan, alginate, Kaltostat, Maxorb, Mesalt comes with sodium chloride, Medi-honey with honey	Heavily exuding ulcer, hemorrhagic ulcer
Hemostatic	Collagen	Cellerate, Fibracol, Prisma, Promogran, puracoll	Traumatic injury, hemorrhagic ulcers
Hydrofibers	Cellulose	Silvercel, Prisma, Aquacel, Promogran, Tegaderm matrix, Dermafill Xylinum Cellulose, Xcell (bacterial cellulose)	Heavily exuding ulcers and infected wounds
Sealants	Dimethicone	Benzoin, Cavilon Barrier Film, Skin-prep, No sting barrier	Puncture wounds, organ wounds
Composite	Multiple types	CombiDERM, Island, Telfa Island, Covaderm plus, Alldress, Dermadress, Adaptic, Adaptic touch, wound veil, Restore, Mepilex, Telfa, CarboFlex, Melolin, Clinisorb, Versiva, Mepitel	Complex wounds needing multiple layers of different dressings

#### Thin Films, Foams, and Wafers

The most commonly used wound dressings are thin flexible sheets of transparent polyurethane with adhesive backing. These dressings are transparent, allowing clinicians to visualize the skin and are also permeable to water vapor, O_2_, CO_2_, but impermeable to bacteria and water. Thus apart from wound healing, they are widely used in sealing the vascular access devices, especially in catheters and saline drip since they are highly elastic and conform to the body contours. The widespread usage of these thin films, impregnated with chlorhexidine, has reduced the incidence of central line infections significantly (Jeanes and Bitmead, 2015). They are also used in superficial wounds, partial-thickness wounds, sutured wounds, donor graft sites, granular wounds, slough-covered wounds with minimal drainage and lacerations or abrasions (Stashak et al., 2004). However, these dressings are ineffective in wounds with high moisture and exudate content since they have minimum absorptive capacity and thus can cause tissue maceration. They should not be used in highly infected wounds and places where the skin is sensitive or fragile because the skin might tear while removing the dressing.

In many cases, excessive exudate secretion is detrimental to the wound healing process. Polyurethane absorptive foam dressings have been developed with a hydrophilic surface to interface with the wound, while the hydrophobic surface faced outside environment. These dressings are permeable to gas, but not to bacteria and other pathogens. Unlike the films, these dressings are highly absorptive and are used in wounds with minimal to heavy exudates, granulating or slough-covered partial-thickness wounds, donor sites, minor burns, diabetic ulcers, and venous insufficiency ulcers (Banks et al., 1997). Foam dressings impregnated with methylene blue have also been used for a bacteriostatic effect (Coutts et al., 2014). The advantages of foam dressings are their ease of use, remarkable absorptive capacity, and availability in various degrees of adhesivity and occlusivity. However, the absorptive aspect of polyurethane foams makes them inappropriate for dry or eschar-covered wounds and arterial ulcers.

Hydrocolloids such as pectin, gelatin, and carboxymethyl cellulose along with adhesives and polymers are used to prepare wafers in thin dressings. These dressings contain hydrophilic colloidal particles with a strong adhesive backing that only need a small area of intact skin to secure, eliminating the need for taping over the dressing (Hutchinson and McGuckin, 1990). These dressings have moderate absorptive capacity but are highly occlusive and are effective barrier against urine, stool, and microbes. Thus, they are used in partial and full-thickness wounds, granular and necrotic wounds, sacral and coccygeal pressure ulcers, minor burns, and venous insufficiency ulcers. However, the hydrocolloidal dressings are contraindicated for heavily draining wounds, infected wounds, arterial ulcers, third-degree burns, and exposed tendons/fascia (Kannon and Garrett, 1995).

#### Glycerin and Alginate Hydrogels

Hydrogels are extensively used in preparing wound dressings. There are many hydrogels in clinical use both in wet and dry (lyophilized) forms. Glycerin-based wound dressings with high water content are available in sheets, gels, or impregnated gauzes (Baum and Busuito, 1998). These are highly moistened and thus absorb minimal amount of fluid but donate moisture to dry wounds. These dressings are permeable to gas and water and are almost always non-adhesive and require secondary bandages. Therefore, these are mainly used for minimally draining wounds, superficial and partial-thickness wounds, softening eschared wounds by moisture and provide padding to decrease shear forces on the wounds (Kirsner, 2016). However, the glycerin dressings are contraindicated in heavily draining wounds and infected wounds.

Another class of hydrogel wound dressings that are widely used is the alginate-based dressings. Alginic acid is extracted from seaweed, converted into sodium salts, and cross-linked with calcium. These dressings are hydrophilic to provide a moist wound environment and are highly absorptive if delivered in a lyophilized form. Since these dressings are highly permeable and non-occlusive, a secondary dressing is needed to keep them in place (Gu et al., 2014). Alginate hydrogels can be fashioned as both sheets for the surface wounds and ropes for the deep wounds. They also are versatile in providing a delivery platform and can be impregnated with silver, honey, and sodium chloride for additional antimicrobial and hyperosmotic properties. Thus, these dressings are used in moderate to highly draining wounds, partial- and full-thickness draining wounds, and infected wounds (Kirsner, 2016). However, they are contraindicated in dry or minimally draining or eschar-covered wounds, arterial ulcers, and exposed deeper structures tendon, joint capsule, or bone.

#### Hemostatics

The most common structural protein in the animal world, collagen, has been used extensively to create hemostatic biosynthetic dressings. The collagen fragments in the dressings induce cell proliferation and chemotaxis while reducing matrix MMP activity (Ruszczak, 2003). MMPs are tissue proteases or endopeptidases that are zinc containing, calcium dependent, and are crucial for wound remodeling phase because they preferentially break down ECM components in the skin (Birkedal-Hansen et al., 1993). Despite the limited studies, and the need for improved study designs and increased number of randomized controlled trials, wound dressings containing collagen appear to have some benefit in the treatment of diabetic foot ulcers and should be carefully considered by clinicians that manage wounds (Holmes et al., 2013). Carboxymethyl cellulose or oxidized regenerated cellulose (ORC) combined with collagen leads to decreased MMP activity, increased cell proliferation, and chemotaxis (Cullen et al., 2002).

#### Composites

To combine the benefits of different kinds of biomaterial dressings, composite dressings have been designed with multiple layers of different biomaterials (Pillay et al., 2015). The bottom or innermost layer is generally composed of a semi or non-adhesive material that allows the wound exudate to flow to the next layer, and it also conforms to the wound’s granulation tissue. This layer is thin, non-adherent/adherent, and non-toxic woven/non-woven mesh. They are often made of polyurethane or polyester, PTFE, and sometimes contain silicone and petroleum complements. They are applied directly to the wound bed and allow the drainage to pass through. The middle layer comprises of highly absorptive material that pulls the wound exudate away from the wound but keeps the environment moist. This reduces skin maceration due to excess moisture and reduces bacterial growth and improves autolytic debridement. The top most or outermost layer is highly occlusive in nature and protects the wound from infection (Wittaya-areekul and Prahsarn, 2006; Elsner et al., 2010). These multilayer dressings can be used as both primary and secondary dressings.

#### Other Biomaterial-Based Wound Dressings

Apart from standard wound dressings, biomaterials have been used to develop skin protectants (Hoggarth et al., 2005), skin sealants (Kemp, 1994), moisture barriers (Zehrer et al., 2005), and keratolytics (Zehrer et al., 2005) that can be delivered as a cream. Skin protectants are often applied to the wound and periwound skin. They prevent maceration of the periwound skin by wound fluid and also prevent rashes and skin breakdown in areas of leakage (Hoggarth et al., 2005). Skin sealants are liquid-based poly-vinyl-methyl (PVM) polymers that form a protective waterproof, breathable, transparent layer on the skin on drying (Kemp, 1994). This protects the periwound skin from moisture, adhesives, and shear stress. Skin sealants work well with adhesive dressing application. The moisture barriers comprise of creams or ointments containing petrolatum, dimethicone, and zinc oxide. Some also contain antifungal miconazole especially useful in treating intertrigo (inflammation of skin folds) (Zehrer et al., 2005). Moisturizers are more hydrophilic creams, lotions, or gels that donate moisture to the wound or periwound skin. Keratolytics soften the hard scales and calluses and hyperkeratotic lesions and include salicylic acid, urea, ammonium lactate creams (Zehrer et al., 2005). However, these keratolytics can cause skin maceration if overused.

## Nanoparticle-Based Wound Therapies

The global nanotechnology industry reached over $1.5 trillion in 2014, becoming a major economic force (Sahoo et al., 2007; Zhou et al., 2014). A central constituent of the nanotechnology industry is engineered NPs. The number of consumer products containing NPs is growing at a rapid pace and is expected to reach 10,000 by the year 2020. NPs have emerged as a new class of therapeutics in the last couple of decades due to their ability to be targeted and low toxicity. NPs are generally defined as particles ranging from 1 to 100 nm in size. These small particles often have different physical and chemical properties from bulk materials. These properties may include alterations in melting points, specific surface areas, specific optical properties, mechanical strengths, and specific magnetizations. These unique properties make them attractive for various industrial and medical applications.

Nanoparticles have become significant in the regenerative medicine field in the last two decades (McLaughlin et al., 2016). Many biological processes happen at through mechanisms that fundamentally act at the nanometer scale. Thus, materials such as NPs can be used as unique tools for drug delivery, imaging, sensing, and probing biological processes (Wang and Wang, 2014). In the context of wound healing, the special properties of NPs like electric conductivity, antimicrobial activity, high surface to volume ratio, swelling, and contraction make NPs versatile resources. In the following sections, we will specifically talk about various NP-based therapeutics that are either undergoing preclinical development or in current clinical use.

### Nanoparticles Currently in Development

The NP-based wound therapies under development are summarized in Table 3.

**Table 3:** **Nanoparticle-based therapies in development**.

Type	Constituent	Therapeutic benefit	Reference
Metal	Silver	Silver nanoparticles enhance wound healing in a full-thickness excisional wound model in mice through the promotion of proliferation and migration of keratinocytes, differentiation of fibroblasts into myofibroblasts	Liu et al. (2010)
	MgF_2_	MgF_2_ nanoparticles effectively restricted biofilm formation of *E. coli* and *S. aureus* by inducing membrane lipid peroxidation and interacting with chromosomal DNA	Lellouche et al. (2009)
	Cerium oxide	Cerium oxide nanoparticles accelerates the healing of full-thickness dermal wounds in mice *via* enhancement of the proliferation and migration of fibroblasts, keratinocytes, and VECs	Chigurupati et al. (2013)
	Copper	Copper nanoparticles-based ointment were twice as good as ointment without copper in healing wounds in mice	Rakhmetova et al. (2010)
	Iron oxide	Thrombin conjugated to iron oxide nanoparticles stabilizes thrombin, increases half-life in body, and enhances wound healing in a rat incisional wound model compared to free thrombin	Ziv-Polat et al. (2010)
	Gold	Spherical nucleic acid gold nanoparticle conjugates efficiently downregulate gene targets in full-thickness wounds in diet-induced obese diabetic mice and fully heals wounds within 12 days whereas control wounds are only 50% closed	Randeria et al. (2015)
Antibiotic loaded	Polyacrylate	N-thiolated beta-lactam antibiotic covalently conjugated onto the polymer framework exhibits potent antibacterial properties against methicillin-resistant *Staphylococcus aureus* and have improved bioactivity relative to free antibiotic	Turos et al. (2007)
	Poly (butyl acrylate–styrene)	Incorporation of a N-thiolated beta-lactam antibiotic onto the nanoparticle matrix endowed the emulsion with antibiotic properties against methicillin-resistant *Staphylococcus aureus*	Garay-Jimenez et al. (2009)
	Chitosan, gelatin, and epigallocatechin gallate	Dressing accelerated mouse wound healing process *via* activated carbon fibers with gentamicin that prevented bacterial infection and nanoparticles that prevented inflammation and facilitated reepithelialization	Lin et al. (2015)
	Folic acid-tagged chitosan	Biocompatible and biodegradable semisynthetic polymer nanoparticles enhance the transport of vancomycin across epithelial surfaces and show its efficient drug action	Chakraborty et al. (2010)
Nitric oxide releasing	Tetramethylorthosilicate, PEG, and chitosan	Nanoparticles increased wound healing by modifying leukocyte migration and increasing tumor growth factor-β production in the wound area, which subsequently promoted angiogenesis	Han et al. (2012)
	Silica	Silica nanoparticles exhibit a 99.999% kill rate against *P. aeruginosa* and *E. coli* and inhibited fibroblast proliferation to a lesser extent than antiseptics like chlorhexidine with proven wound-healing benefits	Hetrick et al. (2009)
Natural Product	Genipin, chitosan, PEG, and silver	Genipin (from *Penicillium nigricans*) cross-linked chitosan, PEG, and silver nanoparticles show high antimicrobial activity against *E. coli*	Liu et al. (2012)
	Silver	Gold and silver nanoparticles synthesized using *Coleus forskohlii* are effective in excisional wound model in albino Wistar male rats	Naraginti et al. (2016)
	Silver	Silver nanocomposite synthesized using *Homalomena aromatica* inhibited the growth of antibiotic-resistant microbes, such as *Staphylococcus aureus, Escherichia coli*, and *Candida albicans*, and fostered wound healing in Wistar rat	Barua et al. (2015)
Lipid based	Proteoliposomes in alginate hydrogel	Improved excisional wound healing and ischemic revascularization *via* enhanced angiogenesis, macrophage modulation, and keratinocyte migration in a diabetic obese mouse model	Das et al. (2016a,b), Monteforte et al. (2016), Tu et al. (2015)
	Solid lipid nanoparticles	Silver sulfadiazine loaded in solid lipid nanoparticles with platelet lysate embedded in chitosan-based dressings showed enhanced wound healing and antimicrobial activity	Gokce et al. (2012), Sandri et al. (2013)
	Exosomes	Human umbilical cord derived MSC exosomes treated wounds exhibited significantly accelerated reepithelialization, with increased expression of CK19, PCNA, and collagen I	Zhang et al. (2015a)
Polymer based	Chitosan, pectin, and titanium dioxide	TiO_2_ nanoparticles loaded in chitosan–pectin scaffolds tested in excisional wound model in albino rats exhibited good antibacterial ability, high swelling properties, excellent hydrophilic nature, biocompatibility, and improved wound closure rate	Archana et al. (2013)
	Hyaluronan	Hyaluronan-based porous nanoparticles encapsulating PDGF-BB was tested in excisional wound healing in rats and showed improved wound healing compared to control	Zavan et al. (2009)

#### Metal Nanoparticles

**Figure 4:**
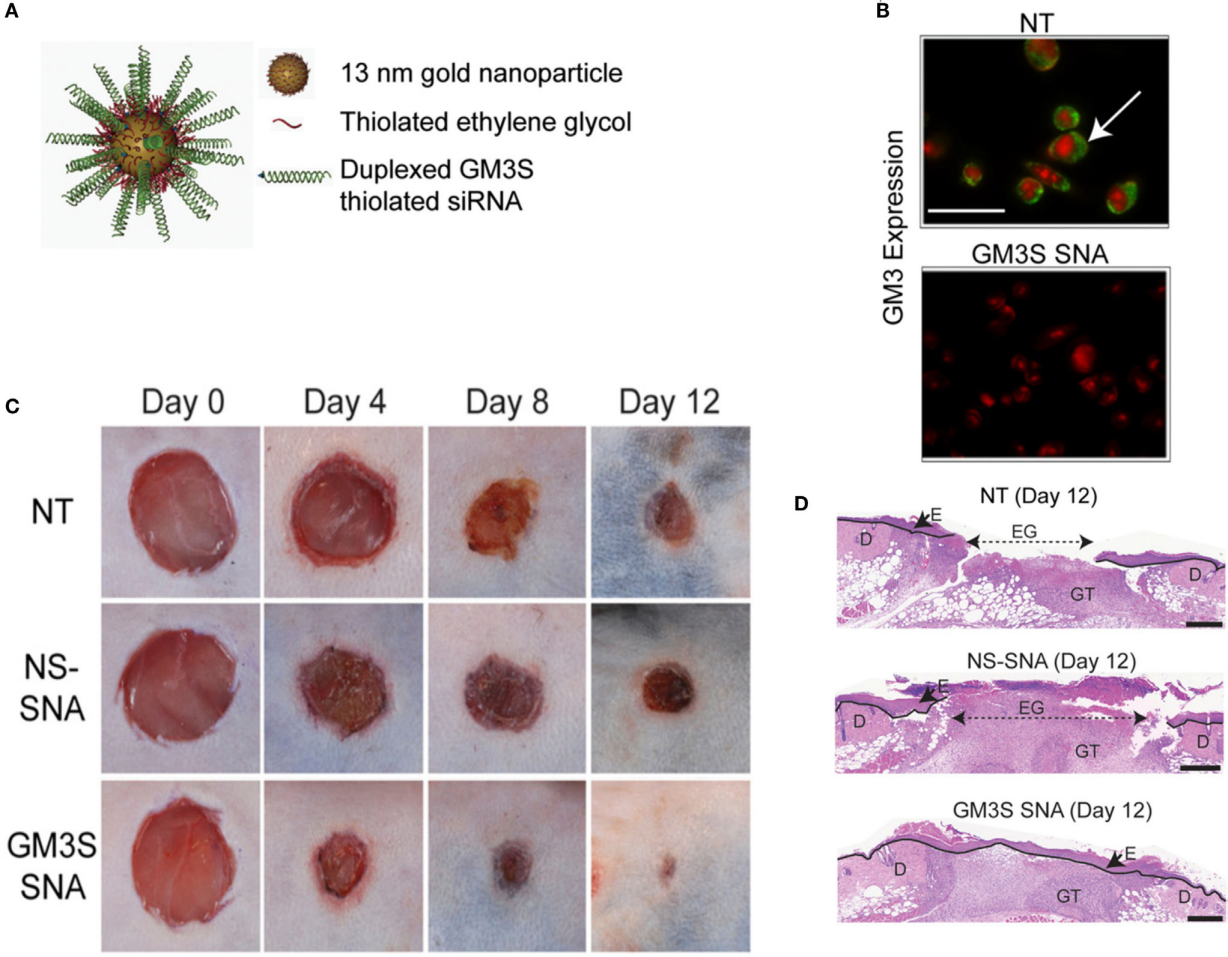
**Overcoming insulin resistance to efficiently heal wounds in a diabetic mouse model**. **(A)** Schematic representation of the gold nanoparticles conjugated to ganglioside–monosialic acid 3 synthase (GM3S) siRNA called the spherical nucleic acid (SNA). The SNA surface is passivated with oligoethylene glycol for colloidal stability. GM3S is a known target that is overexpressed in diabetic mice and responsible for causing insulin resistance and impeding wound healing. **(B)** Confocal images show elimination of GM3 in keratinocytes treated with GM3S SNA (lower) relative to no treatment NT (upper). Green stained GM3; red stained nuclei. Bar = 50 μm. **(C)** Macroscopic clinical images of the wounds in a diabetic diet-induced obesity mouse model over the course of 2 weeks with three different treatments. **(D)** Representative histologic images of the treated wounds at day 12. D, dermis; E, epidermis; EG, epidermal gap; GT, granulation tissue. Bar = 500 μm. NS, non-sense; NT, non-treated. Reproduced with permission from Randeria et al. (2015).

Silver nanoparticles (AgNPs) are the most widely studied among metal NPs. These NPs have been shown to enhance healing in a full-thickness excisional wound model in mice (Liu et al., 2010). Dressings impregnated with AgNPs have also been shown to be effective in wound healing in normal and diabetic mice (Tian et al., 2007). The antimicrobial properties of silver have been exploited in toxicity evaluation in human ASCs (Samberg et al., 2012), human cancer lines (Arora et al., 2008), human keratinocytes (Samberg et al., 2010), and other human cell lines (AshaRani et al., 2009). In addition, AgNPs have also been shown to be anti-inflammatory in a peritoneal adhesion model (Wong et al., 2009). Recently, the safety and efficacy of collagen-coated AgNPs encapsulated in collagen hydrogels was shown in primary human skin fibroblasts and keratinocytes; while antimicrobial properties were shown against *S. aureus, Staphylococcus epidermidis, E. coli*, and *P. aeruginosa* (Alarcon et al., 2015). To gain insight on the health and environmental impact of AgNPs, they were tested on zebrafish models (Asharani et al., 2008) and found that the NPs induce a dose-dependent toxicity in embryos. This may support a non-specific action of AgNPs on all cell types including the wounded host cells. Magnesium fluoride (MgF_2_) NPs (Lellouche et al., 2009) made using the standard microwave method (Jacob et al., 2006) are highly effective against nosocomial microbes including *E. coli* and *S. aureus*. Topical application of water-soluble cerium oxide NPs (Nanoceria) accelerates the healing of full-thickness dermal wounds in mice (Chigurupati et al., 2013). The mechanism of action is thought to be the strong antioxidant properties of cerium oxide NPs. Similarly, copper NPs have also been shown to enhance wound healing in excisional wounds of mice (Rakhmetova et al., 2010). Iron oxide NPs conjugated to thrombin have been used to enhance wound healing compared to free thrombin (Ziv-Polat et al., 2010). This was achieved by increasing the stability of thrombin *via* conjugation to the iron oxide. Gold NPs co-delivered with epigallocatechin gallate and α-lipoic acid significantly accelerated mouse cutaneous wound healing through anti-inflammatory and anti-oxidation effects (Leu et al., 2012). Gold NPs conjugated to siRNA-based spherical nucleic acids (SNAs) have been used for diabetic wounds with ganglioside–monosialic acid 3 synthase (GM3S) knockdown (Randeria et al., 2015). GM3S is an enzyme that is overexpressed in diabetic mice and may cause insulin resistance and reduced wound healing. *In vivo* studies with diet-induced obese diabetic mice showed decreases in local GM3S expression by >80% at the wound edge through an siRNA pathway and fully heals wounds clinically and histologically within 12 days, whereas the control-treated wounds were only about half of the wounds were closed (Figure 4). Gold NPs have also been used with de-cellularized porcine diaphragm as a scaffold for migrating wound cells (Cozad et al., 2011). Among all the metal NPs, we think that the most promising therapeutic options are the gold and silver NPs because of their versatility. While silver is antimicrobial and anti-inflammatory, gold can be easily functionalized for precise delivery of drug or cargo.

#### Antibiotic-Loaded Nanoparticles

There has been a recent surge in advanced therapeutics targeting the multidrug-resistant microbes using antibiotics linked to NPs, commonly referred to as nanobiotics. New classes of polyacrylate NPs that are conjugated to antibiotics were created to treat MRSA (Turos et al., 2007). They consist of water-insoluble N-thiolated beta-lactam antibiotics covalently conjugated to the nanopolymer. These nanobiotics significantly increased antimicrobial activity of the antibiotics in comparison to the non-conjugated antibiotic formulation. Similarly, poly(butyl acrylate-styrene) NPs conjugated to N-thiolated beta-lactam antibiotic have been prepared with conventional and polymerizable surfactants have showed higher antimicrobial activity while maintaining low toxicity (Garay-Jimenez et al., 2009). Gelatin, chitosan, and epigallocatechin gallate NPs have also been incorporated in a polyglutamic acid and gelatin hydrogels containing activated carbon fibers with gentamicin, to create a wound dressing to enhance regeneration and inhibit microbial growth (Lin et al., 2015). Vancomycin-modified NPs produced by magnetic confinement are also highly effective against both Gram-positive and Gram-negative bacteria (Kell et al., 2008). In addition, folic acid-tagged chitosan NPs have been used as “Trojan horses” to deliver vancomycin into bacterial cells and efficiently kill them (Chakraborty et al., 2010). With the rise in antibiotic-resistant bugs, the need for therapies targeting Gram-negative bacteria has become urgent matter, and NP delivery systems may provide a means to enhance the activity of conventional antibiotics in the wound environment.

#### Nitric Oxide Releasing Nanoparticles

Nitric oxide (NO) plays numerous roles in wound healing and can regulate deposition of ECM proteins, cell proliferation, and endothelial function. Incorporation of a functional group of diazeniumdiolate into materials results in the release of biologically active NO when exposed to an aqueous environment (DeRosa et al., 2007). There have been several studies showing the increased wound healing rate due to delivery of NO in a wound microenvironment (Blecher et al., 2012; Han et al., 2012). Biofilms of *P. aeruginosa, E. coli, S. aureus, S. epidermidis*, and *Candida albicans* were formed *in vitro* and exposed to NO-releasing silica NPs, which showed greater than 99% kill rate (Hetrick et al., 2009). It is interesting to note that endogenous NO may actually protect bacteria against antibiotics and other microorganisms (Gusarov et al., 2009). NO-mediated resistance is achieved through both the chemical modification of toxic compounds and the alleviation of the oxidative stress imposed by many antibiotics. Thus, inhibition of bacterial NO synthase might be a suitable future target to enhance antimicrobial therapy.

#### Green Synthesized Nanoparticles

Green synthesis of NPs involves using plant products or extracts that are less expensive and less harmful to the environment than the standard physicochemical methods that are generally used (Makarov et al., 2014). Genipin is prepared from geniposide by using the enzyme β-glucosidase, which is extracted from *Penicillium nigricans*. Genipin cross-linked with chitosan along with PEG and silver NPs are blended into a nanocomposite for enhanced wound healing and high antimicrobial activity (Liu et al., 2012). The formulated silver NPs using *Coleus forskohlii* root extract has been shown to be effective in healing full-thickness excision wounds in albino Wistar male rats (Naraginti et al., 2016). Silver NPs were produced in octadecylamine-modified montmorillonite clay that was mixed with extracts from *Homalomena aromatica* then mixed with hyper branched epoxy to create silver nanocomposite for wound healing applications (Barua et al., 2015). This nanocomposite served as an efficient wound healing scaffold with inherent antimicrobial properties.

#### Lipid Nanoparticles

**Figure 5:**
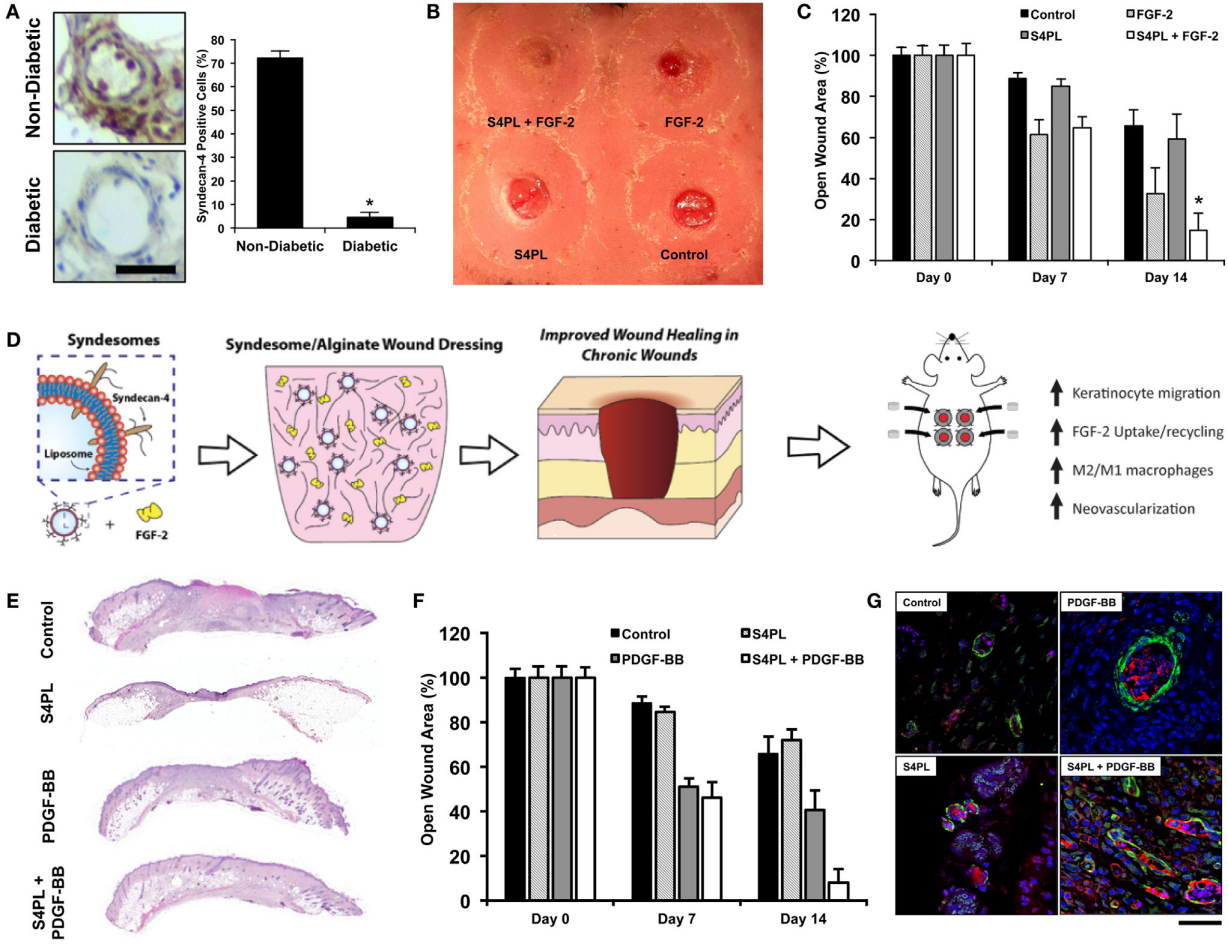
**Delivery of co-receptors with growth factors in a lipid nanoliposome to enhance diabetic wound healing**. **(A)** Protein expression of syndecan-4 in diabetic and non-diabetic human tissue. Bar = 25 μm. **(B)** Macroscopic image of the dorsal surface of the mouse with excisional wounds treated for 2 weeks with the treatments. **(C)** Quantification of the open wound area over the course of 2 weeks after surgery with the different treatments. **(D)** Schematic representation of the co-delivery of syndecan-4 in a nanoliposome with FGF-2 encapsulated in alginate wound dressings and the findings of the study. **(A–D)** are reproduced with permission from Das et al. (2016a). **(E)** Histological sections of wounds with various treatments stained with Hematoxylin and Eosin stain. **(F)** Quantification of the open wound area over the course of 2 weeks after surgery with the different treatments. **(G)** Immunofluorescent images of sections of the wound bed stained with alpha smooth muscle actin (green), PECAM (red), and DAPI (blue). **(E–G)** are reproduced with permission from Das et al. (2016c).

Lipid-based NPs have given rise to an entire subfield of lipid nanotechnology (Mashaghi et al., 2013). Liposomes are versatile drug delivery system due to their ease of protein delivery, biocompatibility, intracellular delivery, modulation of size, charge, and surface properties (Safinya and Ewert, 2012). It has been shown that the loss of growth factor co-receptors in diabetic diseased state leads to growth factor resistance (Das et al., 2014), which may prevent the effectiveness of growth factor treatments to induce angiogenesis and wound healing. One method to overcome this resistance is to deliver co-receptors in a proteoliposome along with the growth factors. This was tested in a diabetic mouse model and showed improved diabetic wound healing (Das et al., 2016a,c) and enhanced ischemic revascularization (Jang et al., 2012; Das et al., 2014, 2016b; Monteforte et al., 2016)(Figure 5). There are various other lipid NPs, which have shown promise for treating peripheral vascular disease and critical limb ischemia, reviewed elsewhere (Tu et al., 2015). Solid lipid nanoparticles (SLN) are a new pharmaceutical delivery system with a solid lipid core stabilized by surfactants, which can solubilize lipophilic molecules. These SLNs have been tested for delivering bioactive molecules such as opioids like morphine (Kuchler et al., 2010b), resveratrol (Gokce et al., 2012), and silver sulfadiazine (Sandri et al., 2013) for wound healing (Kuchler et al., 2009, 2010a). Exosomes are another form of lipid NPs produced by cells and have been shown to be effective for wound healing (Rani and Ritter, 2015).

#### Polymer Nanoparticles

Wound dressings loaded with titanium dioxide NPs that are coated with chitosan and pectin are antimicrobial and have been shown to have great wound healing properties (Archana et al., 2013). The synergistic effects with the dressing such as antibacterial activity, high swelling properties, high moisture vapor transmission rate, hydrophilic nature, biocompatibility, wound appearance, and enhanced wound closure rate make titanium NPs a suitable candidate for wound healing applications. Growth factors are important in ensuring healthy wound healing. However, the half-life of the growth factors in the wound microenvironment is significantly reduced because of the presence of various proteolytic enzymes including MMPs (Murphy and Nagase, 2008). Encapsulation of growth factors in polymer NPs increased stability, preserved bioactivity, and promoted sustained release of the growth factors. Currently, PDGF-BB is the only growth factor that is FDA approved for diabetic foot ulcers, which makes PDGF-based therapies even more translational. Hyaluronan-based porous NPs enriched with PDGF-BB have been shown to be highly effective for the treatment of ulcers in a placebo-controlled study in rats (Zavan et al., 2009). Fibroblast growth factor-2 (FGF-2) has been successfully microencapsulated in gelatin preserving biological activity and thus allows for their use in tissue engineering, therapeutic angiogenesis, gene therapy, and drug delivery applications (Young et al., 2005). EGF is a potent mitogen for keratinocytes, which has been shown to be effective in healing gastric ulcers when delivered through a poly-l-lactic acid-based wound dressing (Han et al., 2001).

### Nanoparticles in Clinical Usage

#### Silver Nanoparticles

Nanoparticle-based therapies in wound care are relatively new compared to conventional biomaterials that have been used for decades now. Silver has been used since ancient Roman times and now used in biomedical devices (de Alwis Weerasekera et al., 2015). Silver NP-based ointments/creams are perhaps the most widely used primarily because of the antimicrobial properties of nanocrystalline silver (Griffith et al., 2015). Silver NPs or nanocrystals in a topical gel have been used for moist wound care and promote cosmetic healing, have effective antibacterial properties, and play a role in cytokine modulation and suppress inflammation (Tian et al., 2007; Jain et al., 2009; Rigo et al., 2013). They are generally indicated for minor cuts, abrasions, lacerations, skin irritations, and first- or second-degree burns. Although the mechanisms underlying the antibacterial actions of silver are still not fully understood, several previous reports showed that the interaction between silver and the constituents of the bacterial membrane caused structural changes and damage to the membranes and intracellular metabolic activity, which might be the cause or consequence of cell death (McDonnell and Russell, 1999; Sondi and Salopek-Sondi, 2004; Pal et al., 2007; Eckhardt et al., 2013). However, prolonged exposure to colloidal silver can result in argyria where the skin attains blue gray color from accumulated silver (Rice, 2009). There are several variations of silver containing creams or gels or ointments that are available from different companies. Silver NP-based treatments are inexpensive, have low systemic toxicity, and are effective against viral and bacterial infections but have limited effects on enhancing the wound healing process in chronic wound environment (Gunasekaran et al., 2011).

## Conclusion

Biomaterials have been used in wound healing since the rise of Egyptian civilization, but NPs have become tremendously important in engineering an effective treatment strategy, only in the last two decades. Biomaterials have been successfully used in manufacturing clinically approved products for aiding wound healing like films, foams, wafers, hydrogels, hemostatics, sealants, and composite dressings. However, there are no biomaterials currently approved that release bioactive components (like growth factors, cytokines, chemokines, plasmids, recombinant proteins, small molecules, cellular therapy, etc.) that directly influence the wound healing cascade. Here, we reviewed biomaterials used in the clinic and those under preclinical development. We are excited about the potential of the biomaterials undergoing development, specifically those that encapsulate bioactive compounds or cell therapies. NP therapies on the other hand have not been used widely in clinic barring silver NPs. However, there is a lot of compelling NP therapies that have shown great potential in animal models as we discussed in the paper.

With the advent of CRISPR-Cas9 technology, it would be interesting to see how scientists apply this remarkable gene editing technology to engineer the wound microenvironment (Jinek et al., 2012; Cho et al., 2013; Cong et al., 2013; Mali et al., 2013; Sander and Joung, 2014). There are many genes that are involved in the regulation of the wound healing process, and wound healing models have been tested only on a few mutant mouse models. CRISPR-Cas9 technology reduces the time to create a knockout mouse from several months to few weeks, thus enabling researchers to ask various questions. The overall goal would be to achieve fetal wound healing properties in adult wound healing with complete regeneration of hairs and glands, without delay and scarring.

Wound care is a significant economic and social burden on both the patient population and the health-care system at large. In this review, we have discussed the different biomaterial and NP-based wound therapies, which are either in current clinical usage or in preclinical development. Since there is significant variability of presentation of symptoms in the patients, effective wound care therapies need to have a multipronged approach to tackle the complex problems of pain, inflammation, infection caused by resistant bacteria, delayed healing, and associated costs to health systems and populations worldwide. The precipitous rise in multidrug-resistant bacteria is going to be the biggest challenge for wound care professionals all over the world in this decade. Emerging treatments using biomaterials or NPs to target multiple aspects have great promise for enhancing wound care and will add to the clinical armamentarium to address poorly healing wounds.

## Author Contributions

SD and ABB wrote and edited the paper.

## Conflict of Interest Statement

The authors declare that the research was conducted in the absence of any commercial or financial relationships that could be construed as a potential conflict of interest.
